# The source of the fat significantly affects the results of high-fat diet intervention

**DOI:** 10.1038/s41598-022-08249-2

**Published:** 2022-03-12

**Authors:** Jiaxing An, Qian Wang, Suqin Yi, Xuemei Liu, Hai Jin, Jingyu Xu, Guorong Wen, Jiaxing Zhu, Biguang Tuo

**Affiliations:** 1 Department of Gastroenterology, Affiliated Hospital of Zunyi Medical University, 149 Dalian Road, Huichuan District, Zunyi, 563003 Guizhou China; 2 Microbial Resources and Drug Development Key Laboratory of Guizhou Tertiary Institution, Life Sciences Institute, Zunyi Medical University, Zunyi, 563006 Guizhou China

**Keywords:** Microbial communities, Experimental models of disease

## Abstract

High-fat diet (HFD) is widely used in animal models of many diseases, it helps to understand the pathogenic mechanism of related diseases. Several dietary fats were commonly used in HFD, such as corn oil, peanut oil, soybean oil, sunflower oil, and lard. However, it was reported that different dietary fat could have completely different effects on physiological indicators and the gut microbiome, and the sources of dietary fat used in high-fat diet research have not been comprehensively compared. In this research, we conduct comparative experiments on various sources of dietary fats to test their different effects during the high-fat diet intervention. We investigated the effects of twelve common dietary fats in high-fat diet intervention of mice, body/liver weight changes, four blood lipid indices, and gut microbiome were analyzed. Our results showed that the source of dietary fat used in high-fat diet significantly affects the changes of body/liver weight and triglyceride (TRIG) in the blood. Furthermore, the intervention of canola oil increased the alpha diversity of gut microbiota, and lard has decreased diversity compared with the control group. The composition of saturated fatty acid (SFA) in fat has the most significant effects on the gut microbiome. All dietary fats treatments have an increasing Firmicutes abundance and a reduced Bacteroidetes abundance in gut microbiome, while the canola oil has a slight variation compared to other intervention groups, and the lard group has the largest changes. This study showed that different types of dietary fat have different effects on the body indicators and intestinal microbiota of mice, and canola oil produced less disturbance than other types of dietary fats in high-fat diet.

## Introduction

Dietary fat is an important part of the diet for humans to obtain lipids and energy. However, it is well known that excessive fat intake could lead to an accumulation of adipose tissue in the body and finally results in obesity and the development of a cluster of metabolic diseases, such as type2 diabetes, atherosclerosis, hypertension, and stroke^1,2^. High-fat diet (HFD) was widely used in animal disease models, including obesity, diabetes, liver disease, and cardiovascular disease. The most typical HFD contained 45–60% of dietary fat as fat source^3–9^. The fats previously used in the HFD researches include soybean oil, corn oil, canola oil, palm oil, lard, etc., and it was widely reported that the gut microbiome is a critical mediator between various diseases and HFD^6,10,11^. The gut microbe has essential roles in supplying nutrients and vitamins, providing colonization resistance against pathogenic bacteria, and interacting with the host immune system and intestinal epithelium^12,13^. However, a few previous reports have shown that different sources of dietary fat have variant effects on obesity and the gut microbiome^14–16^. Except for some small amounts of secondary metabolites and vitamins, the most significant difference between diet fats is the diverse content of three main types of fatty acids, which include saturated fatty acid (SFA), monounsaturated fatty acid (MUFA), polyunsaturated fatty acid (PUFA). It was proved that lard and palm oil, which contain higher SFA, are relatively unhealthy, excessive ingesting of these fats will significantly affect intestinal microbiota and body weight^15^. Conversely, a higher proportion of unsaturated fatty acids in fish oil and olive oil is relatively healthy^17–19^. It indicated that different dietary fats have an unequal contribution to weight gain and gut microbiome changes.

In the last decade, there have been some studies comparing the role of different types of fats in HFD. Studies with palm oil, olive oil, safflower oil and flaxseed/fish oil show that both the type and quantity of fat have different impact on physiology and intestinal microbiota, and the negative effects caused by palm oil was more severe^20^, Palm oil, which is rich in saturated fatty acids compared with olive oil and safflower oil, can induce higher body weight and hepatic triglycerides, and has a greater impact on the diversity of intestinal bacteria^21^. The different effects on gut microbes were also reported between olive oil and butter^19^. Lipidomics-based studies have shown that HFD with fish oil and lard had significantly different effects on hepatic cholesterol metabolism, and fish oil has a lesser effect on hepatic cholesterol^17^. Studies based on Fish oil, lard and soybean oil have shown that fish oil has a different effect on the gut microbiota compared with lard and soybean oil, and can induce the expression of more inflammatory factors, which may have stronger negative effects^22^. On the other hand, it was reported that lard activates WAT inflammation and reduces insulin sensitivity through the TLR signalling pathway^18^. Most of these studies mainly focus on comparative studies of a few types of dietary fat, such as lard, olive oil, fish oil, palm oil, etc., while other fat types, including canola oil, soybean oil, sunflower oil, peanut oil etc., which are widely used in HFD or cooking, are few comparative studied, and the differences between these oils in HFD are unclear.

As such, this study sought to examine two hypotheses on the impact of dietary fats on body indexes (including body weight, liver weight, blood lipid indices) and gut microbiota in HFD. First, we examined the impacts of various types of dietary fat in HFD for exploring whether these fasts have different effects on body indexes. Second, we used 16s rRNA gene sequencing to assess whether dietary fats have different effects on gut microbiota community structure and composition in HFD. Specifically, we assess the impacts of different dietary fats and the contribution of the three main components of fat (include SFA, PUFA, and MUFA) on body indexes and gut microbiota. Together, these studies provide insight into critical information about the different effects of dietary fats on body metabolism and gut microbiome in HFD intervention, and provide some guidance on the choice of fat sources in HFD and the dietary oils in cooking.

## Materials and methods

### Animal experiment and materials

Male C57BL/6 mice (male, 20–22 g) had been bought from the Model Animal Research Center of Nanjing University and been bred in a 12:12 h light–dark cycle, water and standard chow diet were provided *ad-libitum*, the composition of chow diet provided by the manufacturer was showed in Table S1. The mice were randomly divided into 13 groups (n = 10/group) at the 8 wks of age, 5 mice per cage, and mice body mass was recorded. The control group fed with standard chow diet still, and the HFD groups fed with standard chow diet added with an additional 50% dietary fat (mixture of chow diet and dietary oil in a ratio of 1:1, wt/wt), water and food provided ad-libitum. After 8 weeks diet intervention, mice body weight was re-recorded, and feces were collected, blood samples was collected from the retro-orbital sinus into EDTA-coated tubes. Then the mice were sacrificed. All dietary oils were purchased from the online shopping site (www.jd.com), and the components of the dietary oils come from the nutrition facts on the packaging (Table 1). For euthanasia, barbiturate (100 mg/kg) was intraperitoneally injected into each mouse and then followed by cervical dislocation to ensure death.

**Table 1:** The content of SFA, MUFA, PUFA in dietary oil, all data are from the manufacturer’s label on the package.

Dietary oil	SFA (g/100 g)	MUFA(g/100 g)	PUFA(g/100 g)
Sunflower seed oil	11	19	70
Linseed oil	9.1	21.6	65.2
Soybean oil	10–13	20 -25	55–63
Walnut oil	9.5	24	66.5
Corn oil	14.2	29.4	56.3
Sesame oil	15	41	44
Peanut oil	17	50–68	22–28
Canola oil	5–10	70–80	5–10
Olive oil	14	79	6.5
Camellia oil	11	79.5	9.5
Lard	40	50–60	10
Palm oil	50	40	10

This study was conducted in accordance with the ARRIVE guidelines, and the experiment was approved by the Laboratory Animal Ethics Committee of the Affiliated Hospital of Zunyi Medical University (No: KLL-2019-029), all methods were performed in accordance with this permission.

### Serum metabolite assessment

The blood was coagulated overnight and then centrifuged at 12,000 rpm for 10 min, and the upper serum was collected. Total cholesterol (CHOL), triglyceride (TRIG), high-density lipoprotein cholesterol (HDL-C), and low-density lipoprotein cholesterol (LDL-C) were measured using AU5800 Clinical Chemistry Analyzer (Beckman Coulter, Inc.) at the Affiliated Hospital of Zunyi Medical University.

### Feces microbiota assessment

After 8 weeks of dietary oil intervention, faeces were collected, total DNA was isolated with the stool DNA extraction kit (TIANMO BIOTECH, China) using 0.1 g of faecal samples from each mouse. The V4 region of the bacterial 16S rRNA gene was amplified using primers sets 515F (5′-GTGCCAGCMGCCGCGGTAA-3′) and 806R (5′-GGACTACHVGGGTWTCTAAT-3′) with a 12-bp barcode at 5′-end of primer 515R. The PCR reaction (25 μl) consisted of 5 ng of total DNA, 1 Unit of EX Taq (TaKaRa, Dalian, China), 1 × ExTaq buffer, 0.2 mM of each dNTP and 0.4 μM of each primer. Amplification condition consisted of an initial denaturation step of 94 °C for 5 min, followed by 30 cycles of 94 °C for 30 s, 55 °C for 30 s, and 72 °C for 50 s and a final extension at 72 °C for 5 min. Replicate PCR reactions were carried out for each sample, and their PCR products were pooled and subject to 1% agarose gel electrophoresis. The correct size band was excised and purified using SanPrep DNA Gel Extraction Kit (Sangon Biotech, Shanghai, China). Then all PCR products were quantified with Nanodrop and pooled together with an equal molar amount from each sample. And the sequencing sample was prepared using TruSeq DNA kit according to the manufacturer's instruction and then applied to an Illumina Miseq system for sequencing with the Reagent Kit v2 2 × 250 bp at Rhonin Biosciences Co., Ltd. (Chengdu, China).

### Sequencing data processing

Low quality reads, primers, barcode sequences, and chimeric sequences were removed when splitting libraries according to unique barcodes using FASTX-Toolkit (v 0.0.13) (http://hannonlab.cshl.edu/fastx_toolkit/index.html). Operational taxonomic units (OTUs) clustering was performed based on 97% sequence similarity using dada2 method in Qiime2 with default parameters^23^. The representative sequence was annotated using the Greengenes reference library (v13_8). All sequencing data were deposited in GenBank short-read archive with access number PRJNA673002.

### Data analysis

Permutational multivariate analysis of variance (PERMANOVA) was used to test The significant differences between groups by Qiime2. Multiple regression on distance matrices (MRM) was used to calculate the relevance of microbiota diversity and physiological indices by the ecodist package in R. One-way ANOVA was analyzed between control and diet intervention groups using GraphPad (v9.0.0). Statistically significant differences in body weight, liver weight, CHOL, HDL-C, LDL-C between dietary fat intervention groups and control group were determined by the non-parametric Kruskal–Wallis test. Correlations between body weight, liver weight, CHOL, HDL-C, LDL-C, fat components, and gut microbiota were assessed by the non-parametric Spearman’s correlation test, p < 0.05 was considered as statistically significant. Random Forest regression, with a rarefied taxonomy table as input data, was used to regress relative abundances of taxa in the temporal profiles of gut microbiota of all samples, using the command “sample-classifier classify-samples” in qiime2 with—p-random-state 9999 settings. The MetaCyc pathway^24^ were filtered from the output data of PICRUSt analysis which was performed using picrust2 plugin in qiime2, and the LEfSe analysis and image generation was performed in the Huttenhower lab Galaxy server^25^ (https://huttenhower.sph.harvard.edu/galaxy/). The results of PICRUSt were plotted in STAMP software (v2.1.3)^26^.

## Results

### The body weight changes dependent on the source of the dietary fats

**Figure 1:**
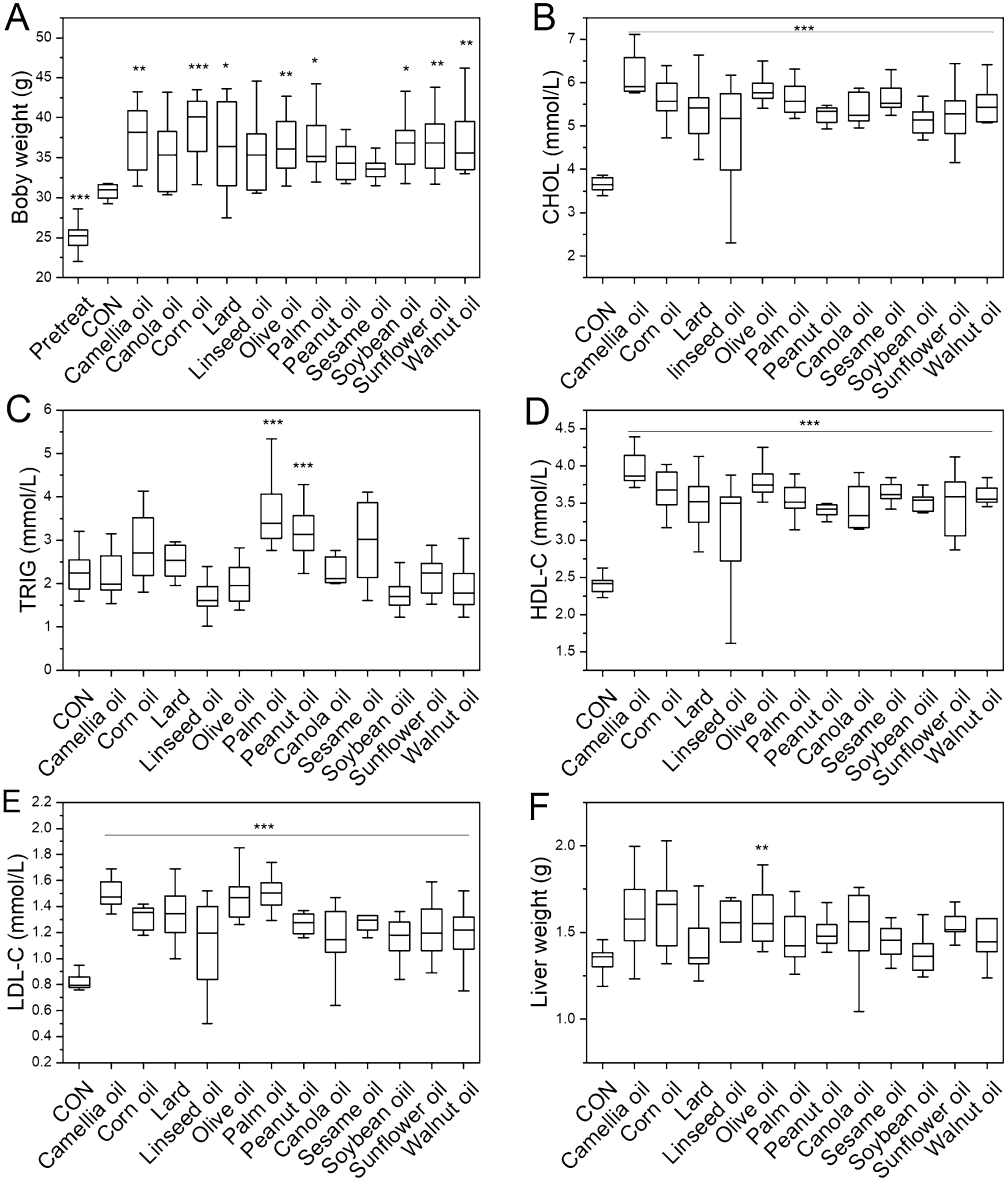
The effects of dietary oil on the body/liver weight and cholesterols of mice. Pretreat, the body weight of mice before intervention at 8 weeks old, and the other groups were measured after 8 weeks oil intervention. All oil intervention groups are compared with the control group. *p < 0.05; **p < 0.01; ***p < 0.001.

To examine whether dietary fats have different effects on the body weight gain of mice, body weight was measured in male mice with different sources of dietary fat intervention. After 8 weeks of dietary fat intervention, the body weight of all HFD diet groups were higher than the control group (30.80 ± 0.93 g), (Fig. 1A), and the corn oil has the highest body weight (38.70 ± 4.06 g), and changes of sesame oil (33.72 ± 1.48 g) was the smallest. The one-way ANOVA results showed that most HFD groups significantly higher body weight (p < 0.05). However, there was no difference in peanut oil (34.48 ± 2.26 g) or sesame oil (33.72 ± 1.48 g) (p > 0.05) intervention groups compared with the control group (30.80 ± 0.93). It implied that peanut oil and sesame oil may not contribute much to weight increase. In contrast, corn oil had the most significant effects on body weight gain. Moreover, the lard (36.38 ± 5.38) had no more weight gain than other oils, and olive oil had a higher effect on weight gain, with a mean body weight of 37.33 ± 5.56 g.

### The HFD induced the increasing of the blood lipid indices

To verify whether different dietary fats have different effects on blood lipids after HFD, triglycerides and three types of cholesterol (CHOL, HDL-C, LDL-C) were surveyed in the serum of mice after the fat intervention. Compared with the control group (3.66 ± 0.16 mM), CHOL concentration in the serum of all HFD groups was significantly higher (Fig. 1B). The top three groups with the most significant changes of CHOL were camellia oil (6.19 ± 0.53 mM), olive oil (5.93 ± 0.45 mM), and palm oil (5.72 ± 0.58 mM), and the types with lower changes were soybean oil (5.04 ± 0.56 mM) and canola oil (5.16 ± 0.81 mM), Among them, the CHOL of lard treatment was not very high (5.40 ± 0.67 mM).

The TRIG content was different between each dietary fats intervention group (Fig. 1C). The groups of lard (2.52 ± 0.12 mM), palm oil (3.66 ± 0.26 mM), corn oil (2.81 ± 0.23 mM), peanut oil (3.19 ± 0.23 mM), canola oil (2.34 ± 0.16 mM), and sesame oil (2.98 ± 0.0.28 mM) had higher levels compared with control (2.28 ± 0.16 mM). Among them, palm oil and peanut oil had the largest increase. In contrast, linseed oil (1.70 ± 0.13 mM), walnut oil (2.00 ± 0.20 mM), camellia oil (2.14 ± 0.16 mM), olive oil (2.05 ± 0.15 mM), soybean oil (1.73 ± 0.12 mM), and sunflower oil (2.18 ± 0.14 mM) groups showed a lower TRIG, of which linseed oil and soybean oil showed larger changes than others.

The content of HDL and LDL in all fat intervention groups was increased significantly (Fig. 1D,E). The camellia oil and palm oil had the most significant increase in HDL and LDL, respectively. In comparison, soybean oil and canola oil had more minor changes in both HDL and LDL after HFD treatment.

### Different effects of dietary fats on liver weight

Compared with the control group (1.34 ± 0.09 g), the liver weight of each treatment group was increased to varying degrees. However, the olive oil intervention group was the highest among all treatments (1.73 ± 0.55 g) (p < 0.05) (Fig. 1F).

The liver index is an index that reflects the health of the liver, which was calculated as liver weight divided by body weight. The results showed that the top highest three groups were olive oil, linseed oil, peanut oil, while only soybean oil treatment was significant lower compared to the control group (p < 0.05) (Fig S1).

### Effects of fat compositions on physiological parameters

To verify the effects of the three main compositions (SFA, MUFA, PUFA) of dietary fat on physiological parameters described above, we analyzed the Pearson correlation between these compositions of fat and physiological parameters. The results indicated that the effects on various physiological indicators showed different trends. Except for MUFA significantly correlates with liver weight, these three fat compositions had no significant correlation with body weight and liver weight (p > 0.05). There was a positive correlation for CHOL, SFA (r = 0.285) and MUFA (r = 0.475), a positive correlation between TRIG and SFA (r = 0.450), and a negative correlation for PUFA (r = − 0.251). For HDL-C and LDL-C, both SFA and MUFA showed a positive correlation (Table 2).

**Table 2:** Correlation analysis between three edible oil components and body weight, liver weight and cholesterol concentration. *p < 0.05; **p < 0.01.

	Body weight	Liver Weight	Liver index	CHOL	TRIG	HDL_C	LDL_C
**SFA**							
Pearson correlation	0.139	0.003	− 0.160	0.285**	0.450**	0.242**	0.424**
p value	0.116	0.975	0.068	0.001	0.000	0.006	0.000
**MUFA**							
Pearson correlation	0.147	0.200*	0.141	0.475**	0.101	0.445**	0.472**
p value	0.094	0.022	0.109	0.000	0.252	0.000	0.000
**PUFA**							
Pearson correlation	0.145	0.006	− 0.155	− 0.011	− 0.251**	0.077	− 0.143
p value	0.100	0.948	0.078	0.902	0.004	0.384	0.105

### Effects of dietary fats on intestinal microbiota

It was reported that the dietary fats intervention truly affected the gut microbiota, this prompted us to investigate whether the structure of the gut microbiota is associated with the sources of dietary fat and changes observed in blood indexes. Therefore, we analyzed the gut bacterial communities after dietary fats intervention using high-throughput sequencing of 16S rRNA gene.

**Figure 2:**
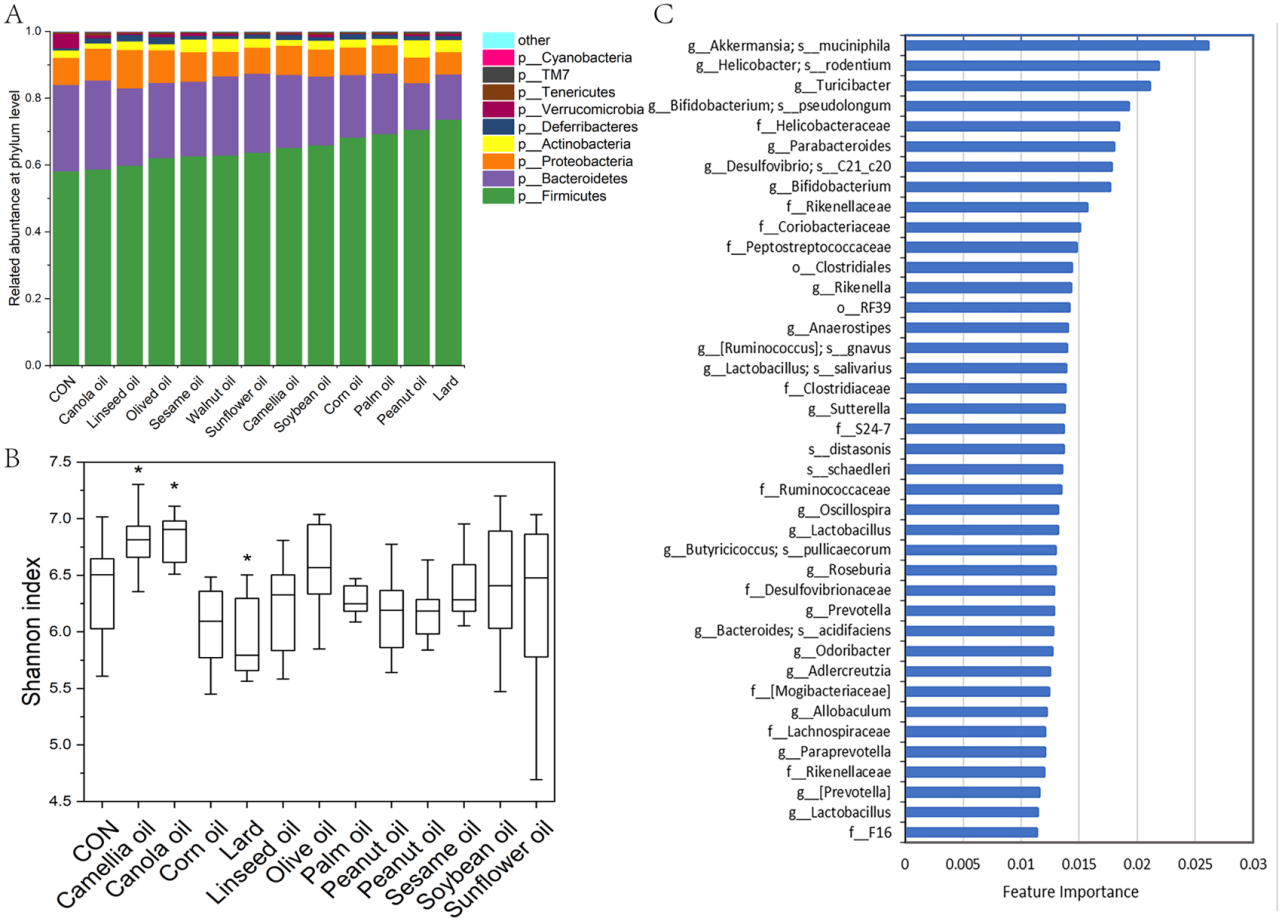
The effects of dietary fats on the gut microbiota. (**A**) The relative abundances of bacterial communities at the phylum level. The relative abundances of the top 9 abundant phyla were shown, while other less abundant phyla and unclassified reads were integrated into others. The treatment groups are sorted in ascending order by the content of Firmicutes. (**B**) The α-diversity indices of Shannon index in different intervention groups. The differences between any two groups were tested by Wilcox test. *p < 0.05. (**C**) Random forest analysis on faecal bacterial communities of all dietary oil intervention groups. The 40 most important predictors were ranked by the Gini index. The taxa are ranked from top to bottom by decreasing Gini index scores.

A total of 122 (8–10 per group) faeces samples were analyzed; after quality control and resampled to 7000 reads per sample, 11,954 OTUs were obtained. The alpha-diversity and beta-diversity were analyzed. The results of Alpha-diversity analysis indicated that the Shannon index of canola oil and camellia oil was significantly higher than the control group. While, the diversity of the lard treatment group was significantly reduced, and other interventions had no difference in the richness of molecular species compare with the control group (Fig. 2B, Fig. S2).

At the phylum level, the three most abundant taxa were Firmicutes, Bacteroidetes, and Proteobacteria (Fig. 2A). Compared with the normal chow group, the Firmicutes of all HFD groups showed an upward trend. The peanut oil and lard intervention groups showed the highest increase, and the changes in canola oil and linseed oil were minor than others. Bacteroidetes showed a downward trend, and it showed almost the opposite trend of Firmicutes in each HFD group, as the relative abundance of lard, peanut oil, and palm oil groups had the lowest richness, meanwhile the canola oil and walnut oil have minor changes. However, the richness of Proteobacteria showed different variation trends between HFD groups, the lard, walnut oil, and peanut oil intervention groups showed declined trends, while the linseed oil, olive oil, and canola oil showed increasing trends. Besides, the abundance of Verrucomicrobia declined in all HFD groups, but the olive oil, canola oil, and soybean oil groups had less decreasing than others. Other phyla did not mention above also showed different abundances change trends under different fat treatments, these results indicated that the sources of dietary fat had significant impacts on the community composition of gut microbiota.

The PERMANOVA based on the Bray–Curtis distance showed significant differences between all 13 groups in this study (p < 0.05). We also analyzed the driving contribution of the gut bacterial community structure, and the results showed that the components of SFA in dietary fat had the most significant effect on it (R^2^ = 0.041) (Table 3). Futher more, we analyzed the correlation between beta-diversity and body indexes in each group, and the results showed that the gut microbiota of linseed oil, sunflower seed oil and sesame oil intervention groups had significant correlation with some body indexes (Table S3). We also generated PCoA plots based on Weighted-Unifrac dissimilarity, but it was hard to cluster each group sample partly because there were too many groups (Fig S3).

**Table 3:** Results of the multiple regression on matrices (MRM) analysis for the whole bacterial community.

	**R**^2^	**Pval**
SFA	0.04140572	0.001
MUFA	0.02655477	0.001
PUFA	0.02229543	0.001
Body weight	0.0003610015	0.524
Liver	0.0002319633	0.744
Liver.w2	0.0004893472	0.577
CHOL	0.022053	0.001
TRIG	0.007180918	0.017
HDL.C	0.02621266	0.002
LDL.C	0.0140675	0.001

**Figure 3:**
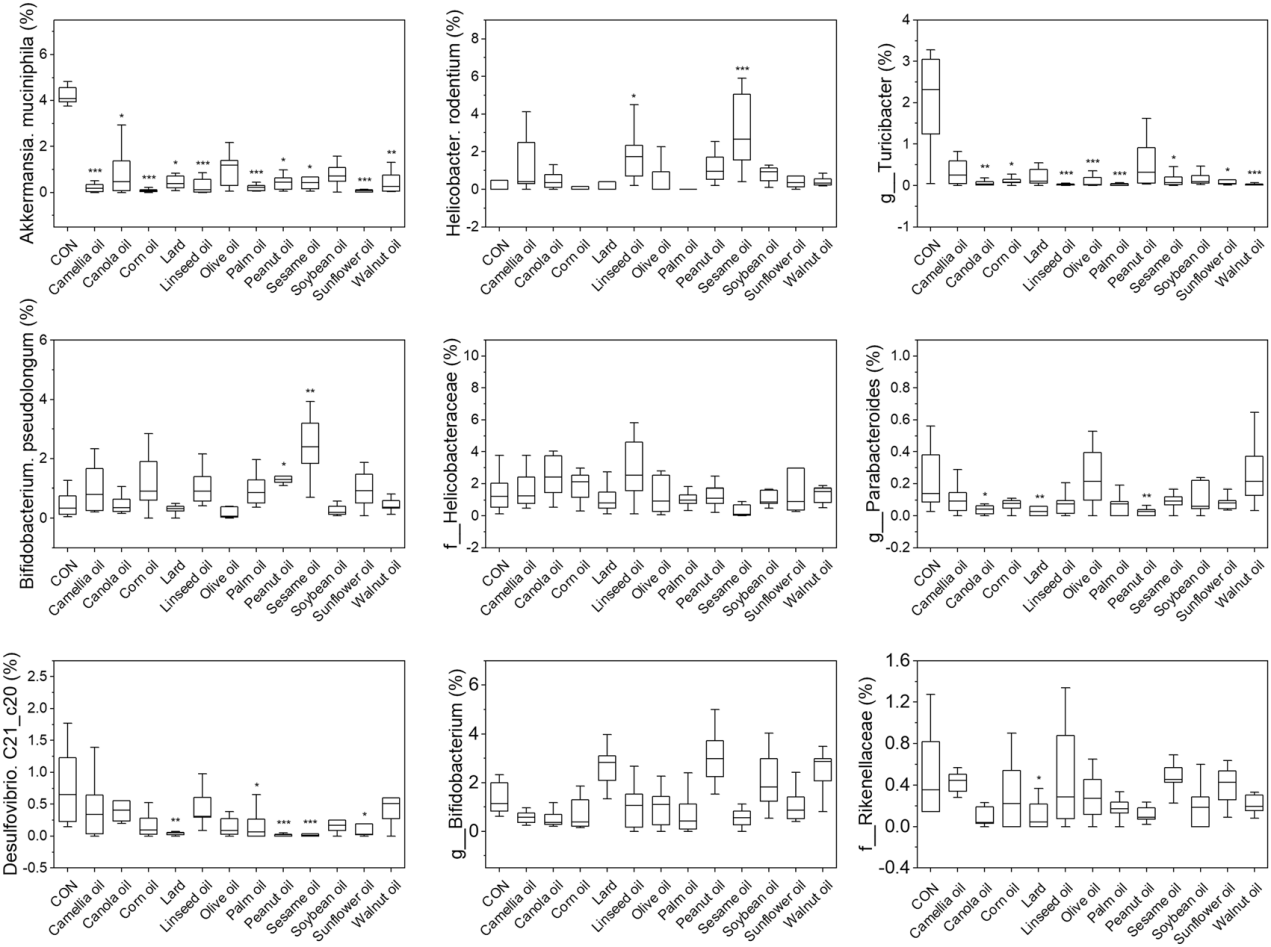
The relative abundance of top 9 importance taxon identified from Random forest analysis in each dietary oil intervention group. All intervention group data were compared with the control group using the Kruskal–Wallis test. *p < 0.05; **p < 0.01; ***p < 0.001.

In addition, we implied the RandomForest cluster to find the important taxonomy for distinguishing each intervention groups, and the results showed the top important five taxa were *Akkermansia muciniphia*, *Helicobacter rodentium*, *Turicibacter*, *Bifidobacterium pseudolongum*, and Helicobacteraceae (Fig. 2C), and their abundance were diversely affected by the source of dietary oils (Fig. 3).

**Figure 4:**
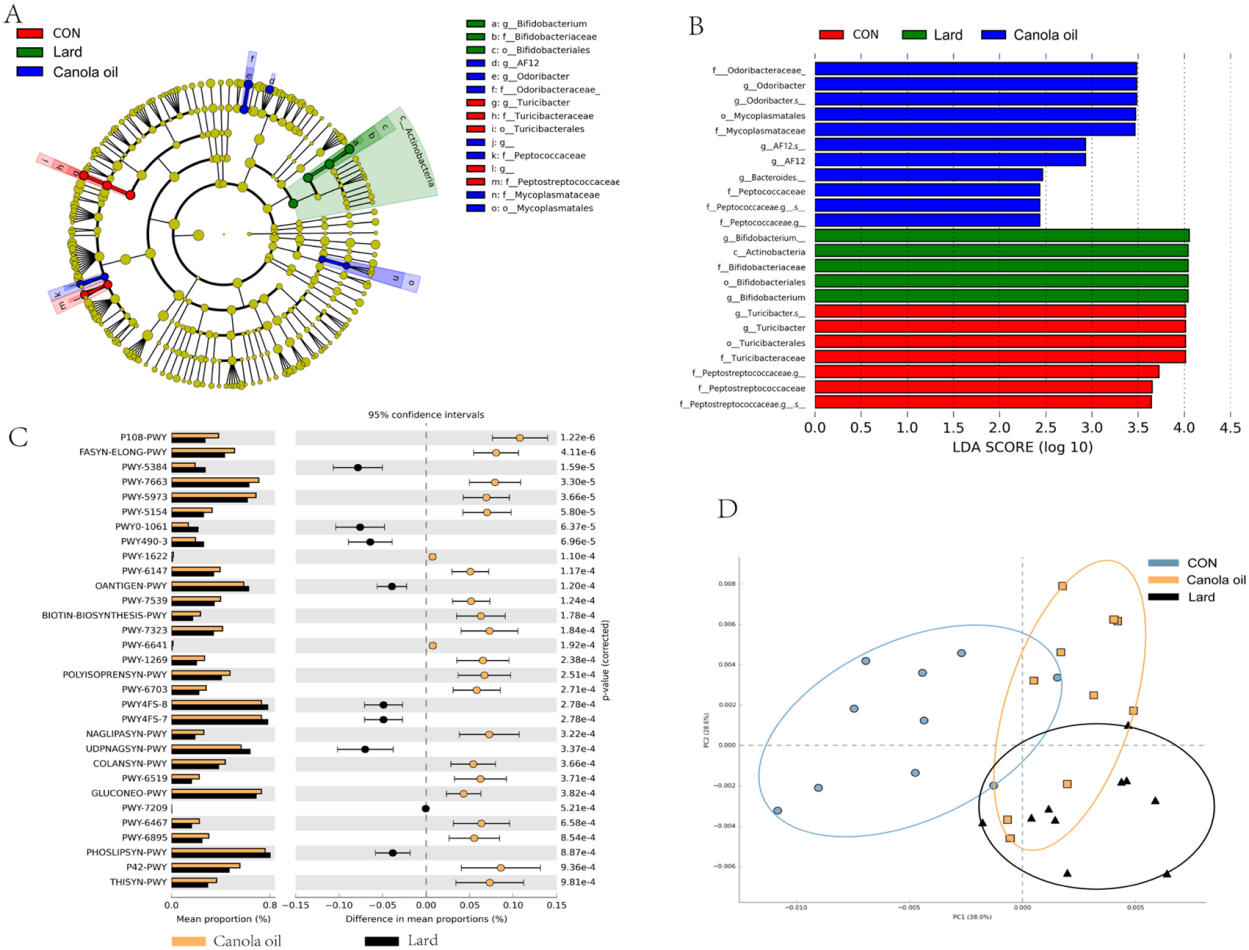
The composition and function changes of microbes between CON, lard, and canola oil intervention group. (**A**,**B**) LEfSe analysis of gut microbiota changes following consumption of high-fat diet intervention. The phylogenetic tree and histogram show LDA score calculated for differences between mice fed different diets, analysis and image generation was performed in the Galaxy webserver. (**C**) PICRUSt prediction of differential MetaCyc pathways in lard and canola oil intervention group based on the 16S rRNA gene sequences. (**D**) PCA plot based on the results of PICRUST analysis.

The gut microbiota results showed the canola oil and lard intervention groups had the biggest difference after HFD treatment. We performed LEfSe analysis to identify taxa that were associated with that are likely to explain differences between control group, canola oil and lard intervention groups. The results showed that the taxa in Odoribacteraceae, Mycoplasmatales, Rikenellaceae, Peptococcaceae, Bifidobacteriaceae, Turicibacter, and Peptostreptococcaceae showed different richness in these three groups (Fig. 4A,B). P108-PWY, FASYN-ELONG-PWY, PWY-5384, PWY-7663, and PWY-5973 were the top five pathways between canola oil and lard (Fig. 4C, Table S2). The PCA analysis based on the PICRUSt analysis showed that the CON, canola oil and lard intervention group were three separate clusters, which indicated that the function of microbes in these groups was very different (Fig. 4D, Fig. S4).

## Discussion

It was widely accepted that the HFD is harmful to health and has an important role in shaping the composition of the gut microbiome, and the HFD was widely used in various animal models of pathogenic mechanism research of many diseases. However, there is no uniform standard for the compositions of feed using in HFD, which may cause different results in HFD experiments in different laboratories. Therefore, we designed this study in light of this situation to investigate the effects of fat sources in HFD on body weight and blood lipids as well as gut microbes to verify whether the fat sources have a significant impact on the experimental results. The result proved that our hypothesis is correct, and this difference does exist and will seriously affect the experimental results of HFD.

As a common animal source of fat, lard is widely used in the HFD experiment of animal models and food cooking of human, many researchers have used lard as a negative control in HFD intervention to highlight the health value of plant-originate fat or fish oil^27–30^. Nevertheless, in this study, although the median value has increased compared with the control group, the body weight increasing of lard intervention group was lower than most of the fat from various plant sources. Moreover, lard effects on live weight, CHOL, TRIG, HDL, and LDL showed similar results. These results indicated that the effects of lard on these indicators are not higher than that of other fats in HFD. Further study of the HFD focus on related indicators, it may need to consider whether to use lard as a negative control carefully.

On the other hand, the impact of lard intervention on gut microbes is much more severe than other fats. The lard intervention had the most significant impact on the abundance of Firmicutes, Bacteroidetes, and Proteobacteria in the intestinal bacteria. We noticed that the composition of SFA in fat was the main driving element of the gut microbiome in this study, it makes sense because lard has a high content of SFA as about 40% in it. However, palm oil, which also has the highest content of SFA, did not show such severe effects on the diversity of the gut microbiome. Because the chemical composition of dietary fat is very complex, especially the oils derived from plants, which contain many kinds of metabolic products, so it is difficult to access the relationship between the diet fat components and the physiological indicators or gut microbiome, it is challenging to clarify the driving mechanism of these components on intestinal bacteria, and it needs further research.

Although almost all HFDs promote weight gain, and HFD cause hepatic steatosis and an increase of inflammatory cytokines in the liver 1^31^, the different content of SFA and UFA can affect some results of HFD. In the study of Caesar et al., HFD with lard, which rich in saturated fatty acids, promotes the obesity process through intestinal bacteria, and promotes the occurrence of WAT inflammation through TLR signaling and CCL2 cytokines. However, fish oil rich in PUSA inhibits this process^18^. Moreover, fish oil does not raise liver cholesterol like lard^17^. This indicates that UFA has protective effects on the metabolism and inflammatory response caused by HFD. UFA mainly contains two types, PUFA and MUFA, and the effects of these two fats on metabolism are also different. Acute ingestion of a PUFA-rich HF meal induced a greater DIT in normal-weight women compared with SFA- or MUFA-rich HF meals^32^. In addition, the two affect the body metabolism at different rates, after a MUFA-rich meal, body show lower RER and greater DIT than PUFA HFD; However, after a 5-d high-fat diet, PUFA diet show greater change in metabolic responses, showing the metabolic adaptability of a PUFA-rich diet^33^. These indicate that the differences in the content of SFA, MUFA and PUFA will significantly affect the results of HFD interference experiments. The types of oils used in this study have great differences in the content of the three main fats, from the results of the correlation between the content of the three fats and body indicators or intestinal microbiota, we can see the differences among these dietary fats.

The correlation analysis between the composition of dietary fat and body weight shows that there is no significant correlation between body weight and the three main components (SFA, PUFA, MUFA). Although we have not counted the amount of food eaten by the mice during the experiment processes, because a considerable part of the feed was wasted during the teeth grinding^34^, we admit that food intake is important data in the HFD experiment, and we indeed observed that the food intake of sesame oil and palm oil treatment groups was slightly less than that of the other treatment groups during the experiment. There are indeed differences in intake between different dietary fat interventions, we also agree that this difference may affect the indicators we measure. However, animal disease models based on HFD usually takes a long time (several months commonly) to induce the corresponding disease occurrence. Therefore, in the animal model experiment of HFD, it is unrealistic to control the intake of animals precisely, and most of them adopt free-eating. Our experimental method was designed according to the general experimental process of HFD, and we believe that such results can guide the choice of fat types for HFD. Regarding the influence of the same calorie intake on HFD, and the influence of specific molecular components in fats on HFD, more research is needed in the future to explain. The blood lipids contents are important indicators for clinical diagnosis, and the raising of blood lipids will significantly increase the probability of suffering from cardiovascular disease^35–37^. We tested four important blood lipid indicators (CHOL, TRIG, HDL, LDL) and found that the different sources of dietary fat have different effects on these indicators. The results of olive oil treatment are not consistent with popular opinions that olive oil has always been considered a healthy dietary fat^38^. This may be because the fat concentration used in our experiment was too high, previous research shows that the effect of fat on the body is concentration-dependent. The analysis results from canola oil intervention data showed that the greatest benefits occurred when ~ 15% of the total energy intake was consumed^39^. It indicated that excessive intake of olive oil could also produce unhealthy results as the other dietary fats, and it also reminds us that when conducting HFD experiments, the fat ratio may also be a critical factor that affects the results of the experiment.

Many studies proved that HFD affects the development of many diseases through the gut microbiome^40,41^. Our results showed that the sources of dietary fat could affect both the composition and diversity of the gut microbiota after HFD intervention. It is noteworthy that intervention with camellia oil and canola oil can increase the diversity of the gut microbiome, and in contrast, lard reduces it. Regarding the effects of a HFD, this difference is sufficient to produce the opposite conclusion. Therefore, in the HFD experiment, the source of the fat used should be carefully considered.

We noticed that canola oil does not seem to affect intestinal bacteria after the HFD intervention, and the body/liver weight or bloody lipid indicators data also showed canola oil has milder effects than other dietary fats. The previous reports show that canola oil can decrease triglyceride, post-intervention grading of fatty liver was reduced significantly^42^; canola oil containing omega-3 PUFAs may confer cardiovascular protection by improving endothelial function and lowering LDL-cholesterol^43^. Meta-analyses results also show that canola oil potentially improves cardiovascular risk factors compared with saturated fat, sunflower, and olive oil^39^. Although excessive intake of fats and oils is generally harmful to health, using canola oil in cooking may minimize the risk of harm. Of course, this result is unfavourable for most HFD experiments, but on the other hand, this has important reference significance for choice of dietary fats for cooking. Although it is not rigorous to conclude that the health value of canola oil is better than other categories based on the data of this research, it can at least partially explain that it is a better choice, and more comprehensive nutrition research should carry out in the future.

We also noticed that the abundance of *Akkermansia muciniphila* changed significantly after HFD treatment, which indicated that it is very sensitive to HFD. *A. muciniphila* is a common human gut bacterium, promotes intestinal barrier function and produces nutrients that feed other gut bacteria^44,45^. It has been getting much attention because several studies have linked *A. muciniphila* to many metabolic and disease development processes, such as obesity^46^, type 2 diabetes^47^, heart disease^48^, SCFAs production^49^, Crohn’s disease^50^, Ulcerative Colitis^51^, Appendicitis^52^. We found that the abundance of *A. muciniphila* was negatively correlated with CHOL, HDL-C, and LDL-C in mice with dietary fat intervention, high-lighting *Akkermansia* as a potential mediator of the improved body mass and bloody lipids phenotype of mice with dietary intervention. This finding is in agreement with previous findings linking *A. muciniphilia* with protection to diet-induced obesity^53^. Even we can not conclude that the microbiota of canola oil intervention is more healthy than lard intervention group without further researches evidence, but the Firmicutes:Bacteroidetes of canola oil group is the smallest among the groups, and the *A. mucinphila* of canola oil group is higher than most others, that indicated that canola oil might be a healthy dietary fat for cooking from our bacterial community data.

Previous studies have shown that Helicobacteraceae increases in mice and humans after consumption of diets rich in saturated fats of animal origin, *Helicobacter* species could infect organs of the gastrointestinal tract, including stomach, intestine, and liver^54,55^*. Helicobacter rodentium* can trigger the development of IBD in mutated mice^56^. Our results showed that dietary oil intervention increasing the abundance of *Helicobacter*, it was closely related to *H. pylori*, which infections can cause severe stomach diseases in human^57–59^. It implied that HFD might increase the risk of *H. pylori* infection.

We compared the metabolic pathways of canola oil and lard groups, and found that of the 31 metabolic pathways with the most significant differences (p < 0.001), 22 of them were up-regulated in rapeseed oil, while only 9 were up-regulated in lard. This may be due to the higher diversity of intestinal bacteria treated with canola oil. Most of the up-regulated pathways are related to fatty acid synthesis and energy metabolism, indicating that the intestinal bacteria treated with canola oil can synthesize more fatty acid-related products, which has certain benefits to the host. While the lard treated group promotes the generation of some harmful substances, such as PWY490-3, promote the reduction of nitrate to nitrite, and excessive nitrite is harmful to the host; and the OANTIGEN-PWY pathway, which is included in the synthesis of O-antigens, and O-antigens is the part of the LPS that can induce some immune responses or cause immune diseases. Although the specific metabolic pathways corresponding to microbes are still unclear, the difference between the microbes and the metabolic pathway can be predicted to determine the corresponding regulatory relationship between them, thus laying a foundation for further study on dietary fats in HFD or cooking.

## Conclusions

The effects of the HFD intervention on mice showed that the source of dietary fat has apparent differences in the effects of body/liver weight, blood lipids and gut microbiome. Especially the gut microbiome is particularly sensitive to the different types of dietary fat, which results in considerable changes in the specific taxon. Furthermore, the composition of SFA in fat is the most significant element that sharpens the gut microbiome community. The HFD intervention induced an increased abundance in all the tested groups of Firmicutes and a decrease in Bacteroidetes and the Verrucomicrobia. The intervention of canola oil has a minor impact on the gut microbiome, while lard has the most significant impacts, which results in differences in microbes composition and metabolic functions. These results suggest that we need to carefully consider the source of fat in HFD researches. On the other hand, canola oil has milder effects on body weight, physiological indicators, and the gut microbiome, which indicated it would be a better choice for daily cooking.

## Supplementary Information

Supplementary Information.

## Data Availability

The sequence data of this study can be found in NCBI with the bioproject number PRJNA673002 or website https://www.ncbi.nlm.nih.gov/bioproject/PRJNA673002.
