# Supplementary material for: The source of the fat significantly affects the results of high-fat diet intervention
Source: Sci Rep. 2022 Mar 12;12:4315. doi: 10.1038/s41598-022-08249-2 (PMC8918335; doi:10.1038/s41598-022-08249-2)
Supplement: Supplementary file 1 — Supplementary Information. [file 41598_2022_8249_MOESM1_ESM.docx]

Table S1. Compositions of the base diet used in the present study (wt/kg).

| Compositions | wt/kg |  | Compositions | wt/kg |
| --- | --- | --- | --- | --- |
| Crude protein | ≥180 g |  | Vitamin K | ≥ 3.0 mg |
| Crude fat | ≥ 40 g |  | Vitamin B1 | ≥ 8.0 mg |
| Crude fiber | ≤ 50 g |  | Vitamin B2 | ≥ 10 mg |
| Crude ash | ≤ 80 g |  | Vitamin B6 | ≥ 6.0 mg |
| Moisture | ≤ 100 g |  | Niacin | ≥ 45 mg |
| Ca | 10-18 g |  | Pantothenic acid | ≥ 17 mg |
| P | 6-12 g |  | Folate | ≥ 4.0 mg |
| Lysine | ≥ 8.2 g |  | Biotin | ≥ 0.1 mg |
| Methionine + Cystine | ≥ 5.3 g |  | Vitamin B12 | ≥ 0.02 mg |
| Arginine | ≥ 9.9 g |  | Choline | ≥ 1250 mg |
| Histidine | ≥ 4.0 g |  | Vitamin C | - |
| Tryptophan | ≥ 1.9 g |  | Mg | ≥ 2.0 g |
| Phenylalanine + Tyrosine | ≥ 11 g |  | K | ≥ 5.0 g |
| Threonine | ≥ 6.5 g |  | Na | ≥ 2.0 g |
| Leucine | ≥ 14.4 g |  | Fe | ≥ 100 mg |
| Isoleucine | ≥ 7.0 g |  | Mn | ≥ 75 mg |
| Valine | ≥ 8.4 g |  | Cu | ≥ 10 mg |
| Vitamin A | ≥ 7000 IU |  | Zn | ≥ 30 mg |
| Vitamin D | ≥ 800 IU |  | I | ≥ 0.5 mg |
| Vitamin E | ≥ 60 |  | Se | ≥ 0.1-0.2 mg |

Table S2. The top 31 pathway identified by PICRUSt analysis between canola oil and lard intervention group.

| BioCyc ID | Pathway name | p-values | p-values (corrected) |
| --- | --- | --- | --- |
| P108-PWY | pyruvate fermentation to propanoate I | 1.22E-06 | 1.22E-06 |
| FASYN-ELONG-PWY | fatty acid elongation -- saturated | 4.11E-06 | 4.11E-06 |
| PWY-5384 | sucrose degradation IV (sucrose phosphorylase) | 1.59E-05 | 1.59E-05 |
| PWY-7663 | gondoate biosynthesis (anaerobic) | 3.30E-05 | 3.30E-05 |
| PWY-5973 | cis-vaccenate biosynthesis | 3.66E-05 | 3.66E-05 |
| PWY-5154 | L-arginine biosynthesis III (via N-acetyl-L-citrulline) | 5.80E-05 | 5.80E-05 |
| PWY0-1061 | superpathway of L-alanine biosynthesis | 6.37E-05 | 6.37E-05 |
| PWY490-3 | nitrate reduction VI (assimilatory) | 6.96E-05 | 6.96E-05 |
| PWY-1622 | formaldehyde assimilation I (serine pathway) | 0.000110158 | 0.000110158 |
| PWY-6147 | 6-hydroxymethyl-dihydropterin diphosphate biosynthesis I | 0.000116953 | 0.000116953 |
| OANTIGEN-PWY | O-antigen building blocks biosynthesis (E. coli) | 0.000120092 | 0.000120092 |
| PWY-7539 | 6-hydroxymethyl-dihydropterin diphosphate biosynthesis III (Chlamydia) | 0.000123694 | 0.000123694 |
| BIOTIN-BIOSYNTHESIS-PWY | biotin biosynthesis I | 0.000178104 | 0.000178104 |
| PWY-7323 | superpathway of GDP-mannose-derived O-antigen building blocks biosynthesis | 0.000184158 | 0.000184158 |
| PWY-6641 | superpathway of sulfolactate degradation | 0.000191971 | 0.000191971 |
| PWY-1269 | CMP-3-deoxy-D-manno-octulosonate biosynthesis | 0.000237735 | 0.000237735 |
| POLYISOPRENSYN-PWY | polyisoprenoid biosynthesis (E. coli) | 0.000251454 | 0.000251454 |
| PWY-6703 | preQ0 biosynthesis | 0.000270937 | 0.000270937 |
| PWY4FS-7 | phosphatidylglycerol biosynthesis I (plastidic) | 0.000278277 | 0.000278277 |
| PWY4FS-8 | phosphatidylglycerol biosynthesis II (non-plastidic) | 0.000278277 | 0.000278277 |
| NAGLIPASYN-PWY | lipid IVA biosynthesis (E. coli) | 0.000322396 | 0.000322396 |
| UDPNAGSYN-PWY | UDP-N-acetyl-D-glucosamine biosynthesis I | 0.000336665 | 0.000336665 |
| COLANSYN-PWY | colanic acid building blocks biosynthesis | 0.000365712 | 0.000365712 |
| PWY-6519 | 8-amino-7-oxononanoate biosynthesis I | 0.000370627 | 0.000370627 |
| GLUCONEO-PWY | gluconeogenesis I | 0.000382342 | 0.000382342 |
| PWY-7209 | superpathway of pyrimidine ribonucleosides degradation | 0.000521333 | 0.000521333 |
| PWY-6467 | Kdo transfer to lipid IVA III (Chlamydia) | 0.00065764 | 0.00065764 |
| PWY-6895 | superpathway of thiamine diphosphate biosynthesis II | 0.000853932 | 0.000853932 |
| PHOSLIPSYN-PWY | superpathway of phospholipid biosynthesis I (bacteria) | 0.00088713 | 0.00088713 |
| P42-PWY | incomplete reductive TCA cycle | 0.000935784 | 0.000935784 |
| THISYN-PWY | superpathway of thiamine diphosphate biosynthesis I | 0.000981419 | 0.000981419 |
|  |  |  |  |


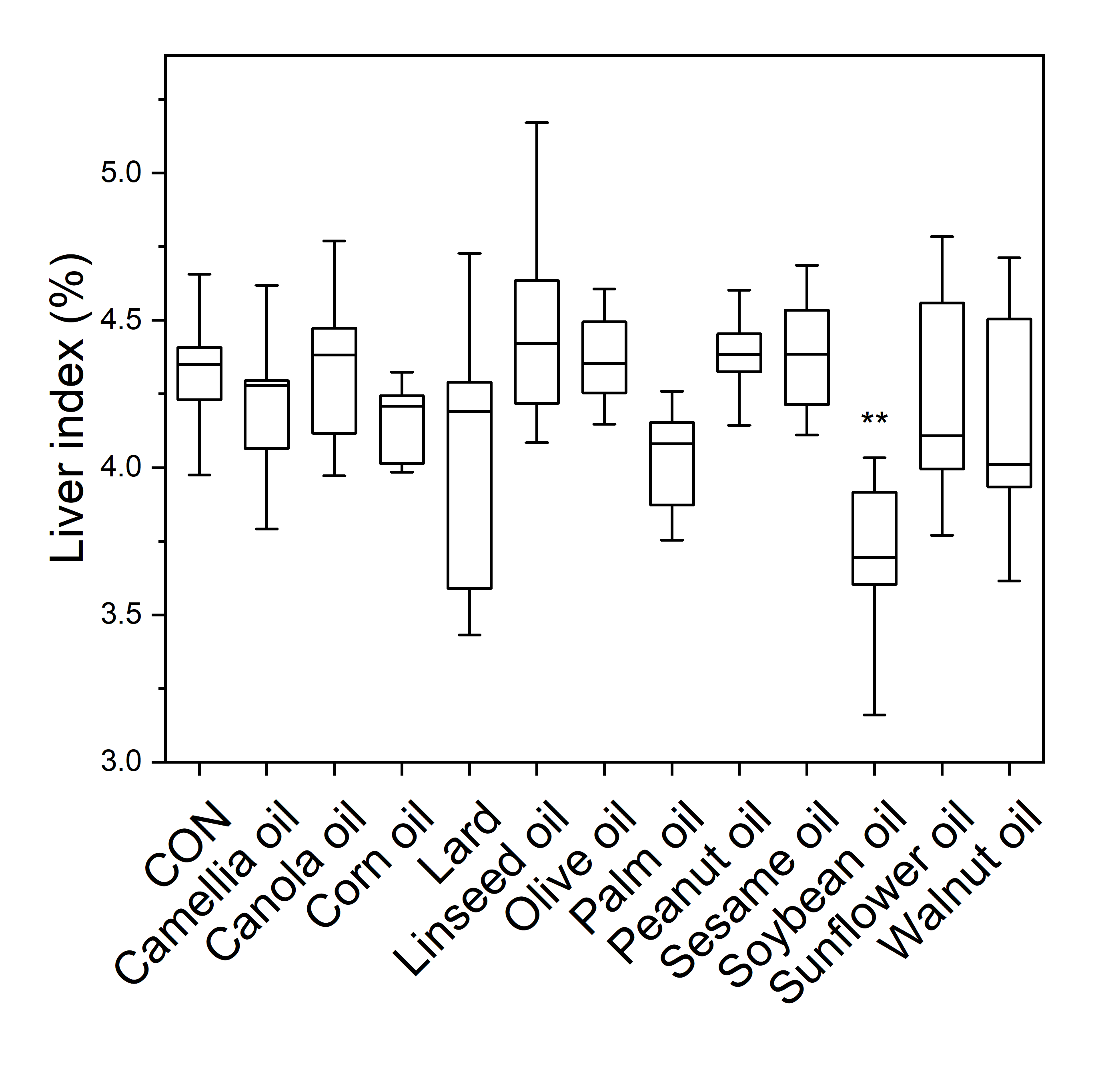


Fig S1. The effects of dietary fat on the liver index of mice. Liver index was calculated by the following formula: Liver index (%) = weight of liver/weight of body x 100%. All intervention groups data are compared with the control group. *, p < 0.05; **, p < 0.01.


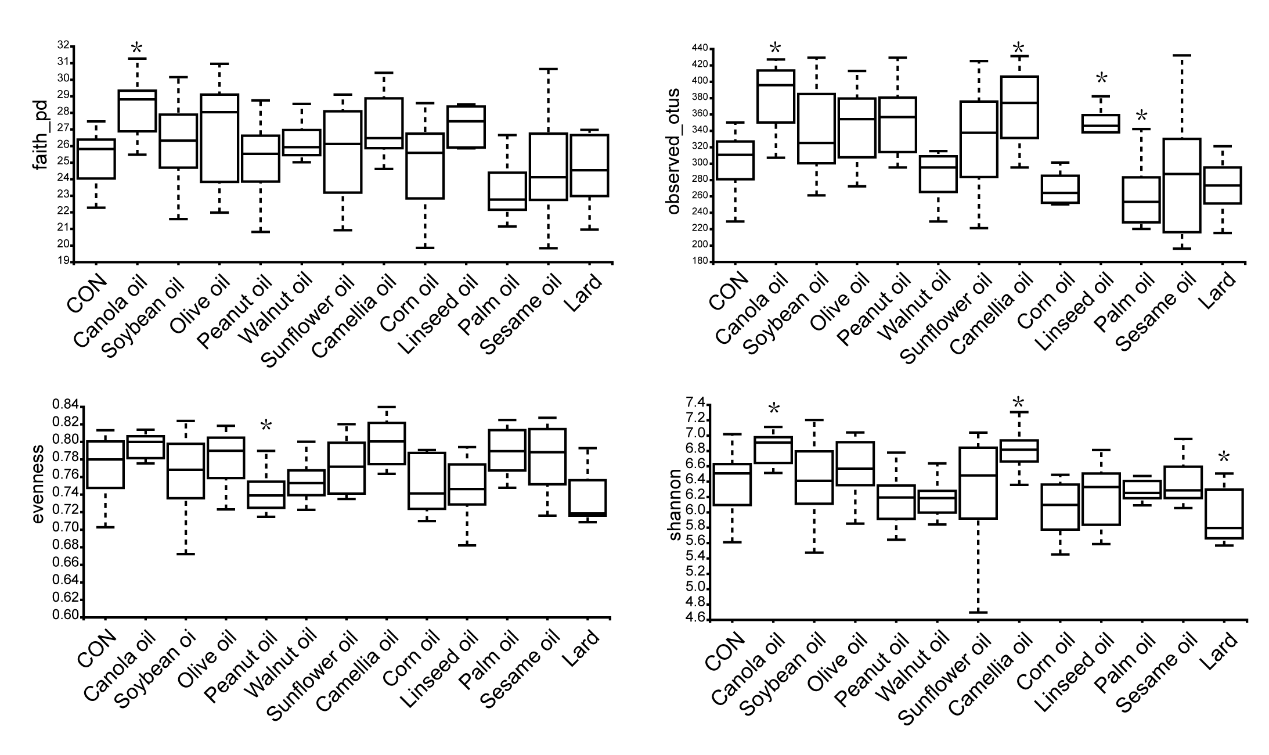


Fig S2 , Alpha diversity of gut microbe between each HFD group. All intervention groups data are compared with the control group. *, p < 0.05.

**
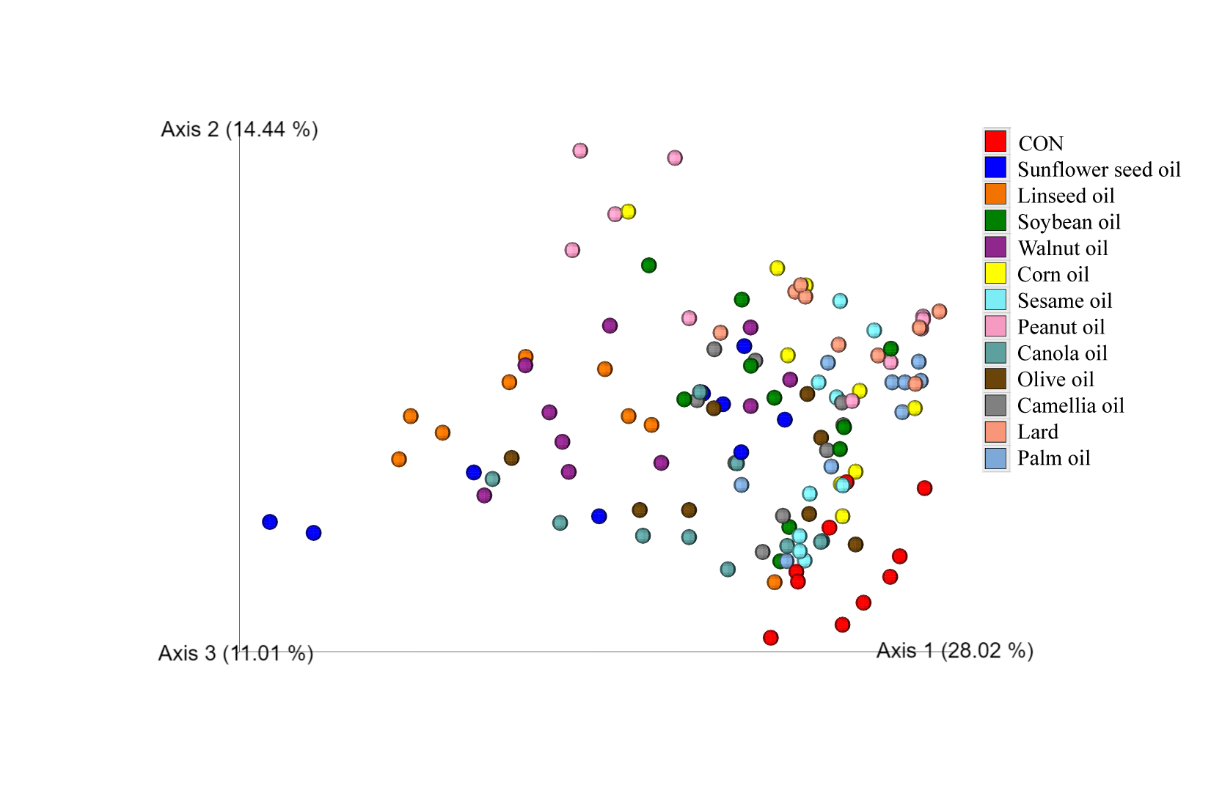
**

Fig S3. PCoA plot based on the Weighted-Unifrac dissimilarity.


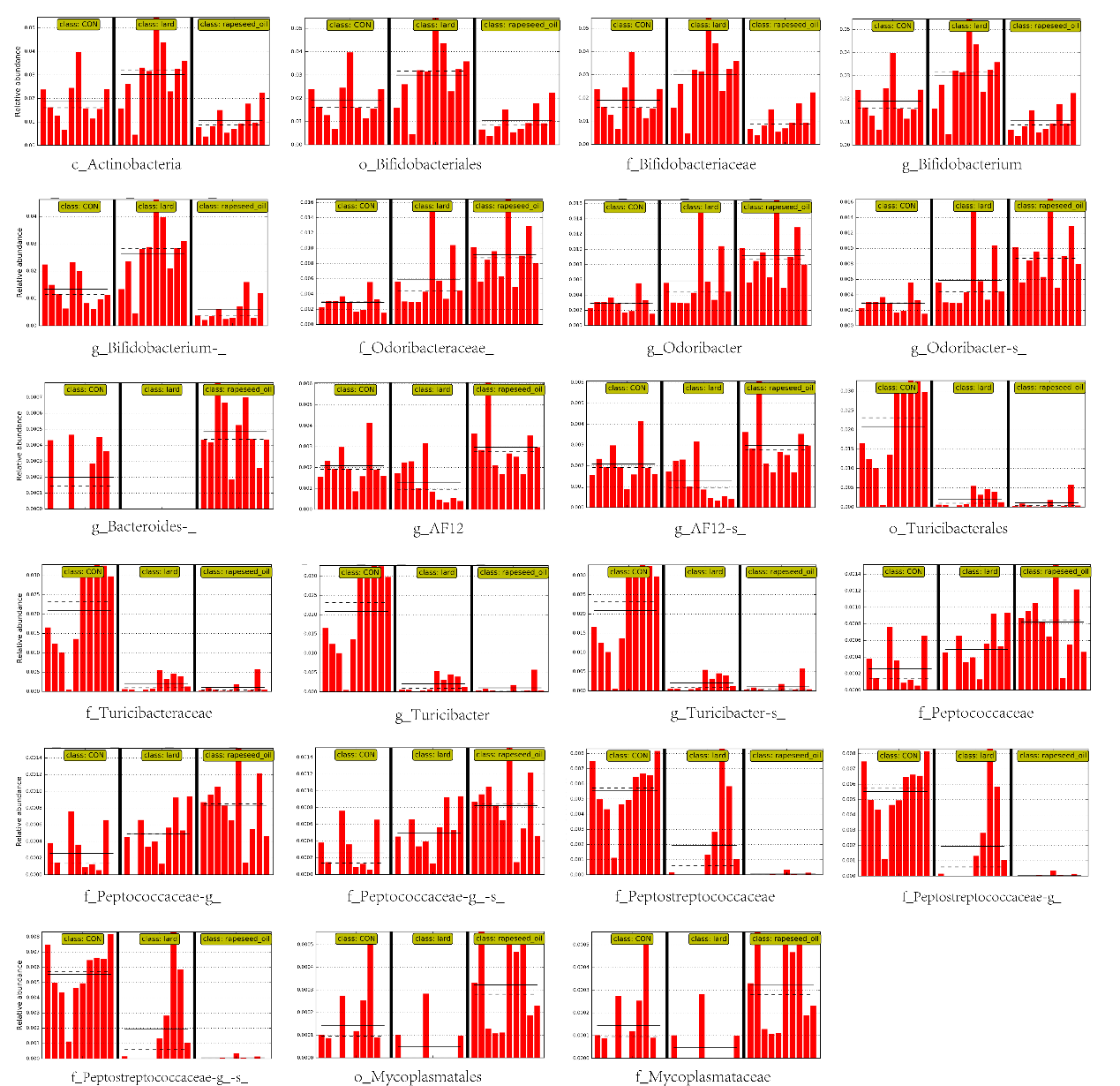


Fig S4. The relative abundance of taxon identified by LEfSe in CON, lard, and canola oil intervention group.

**Table S3.** Correlation analysis between the microbiota and body weight, liver weight and cholesterol concentration in each HFD group, mantel test was analyzed with Bray-Curtis distance of microbiota of each group.

|  |  | weight2 | liverW | liver.W2 | CHOL | TRIG | HDL.C | LDL.C |
| --- | --- | --- | --- | --- | --- | --- | --- | --- |
| CON | Mantel coefficient | -0.24294 | -0.0394 | -0.09157 | -0.10761 | 0.112806 | 0.153643 | -0.00265 |
|  | p-value | 0.989 | 0.56 | 0.681 | 0.763 | 0.243 | 0.171 | 0.476 |
| Camellia oil | Mantel coefficient | 0.140218 | 0.162677 | 0.073616 | 0.047113 | 0.202626 | 0.086279 | 0.176638 |
|  | p-value | 0.194 | 0.189 | 0.277 | 0.308 | 0.107 | 0.224 | 0.147 |
| Olive oil | Mantel coefficient | 0.123153 | 0.247948 | 0.070608 | 0.26006 | -0.08539 | 0.073932 | -0.03917 |
|  | p-value | 0.267 | 0.19 | 0.359 | 0.178 | 0.632 | 0.336 | 0.541 |
| Soybean oil | Mantel coefficient | 0.188089 | 0.322738 | -0.1137 | 0.0446 | -0.19551 | 0.127512 | -0.08513 |
|  | p-value | 0.144 | 0.062 | 0.727 | 0.396 | 0.876 | 0.269 | 0.662 |
| Sunflower seed oil | Mantel coefficient | -0.11818 | 0.018445 | 0.274835 | 0.293959 | -0.10214 | **0.367434** | 0.117418 |
|  | p-value | 0.766 | 0.408 | 0.067 | 0.05 | 0.74 | **0.025** | 0.22 |
| Lard | Mantel coefficient | 0.102587 | -0.3565 | -0.06821 | -0.05058 | 0.005149 | 0.080973 | 0.059358 |
|  | p-value | 0.298 | 0.975 | 0.642 | 0.609 | 0.454 | 0.384 | 0.374 |
| Palm oil | Mantel coefficient | -0.18072 | -0.2111 | 0.191144 | -0.01474 | 0.172503 | 0.35357 | 0.287251 |
|  | p-value | 0.885 | 0.893 | 0.121 | 0.443 | 0.188 | 0.063 | 0.09 |
| Corn oil | Mantel coefficient | 0.069755 | -0.05483 | -0.06229 | -0.00772 | 0.115466 | -0.02934 | -0.24361 |
|  | p-value | 0.281 | 0.648 | 0.646 | 0.496 | 0.211 | 0.526 | 0.954 |
| Peanut oil | Mantel coefficient | -0.1151 | -0.00448 | 0.180249 | -0.08491 | 0.153633 | 0.016159 | -0.04339 |
|  | p-value | 0.788 | 0.451 | 0.08 | 0.745 | 0.132 | 0.446 | 0.603 |
| Linseed oil | Mantel coefficient | **0.506662** | 0.051355 | -0.10528 | 0.052069 | **0.499775** | 0.045261 | 0.086176 |
|  | p-value | **0.001** | 0.318 | 0.596 | 0.315 | **0.027** | 0.337 | 0.258 |
| Canola oil | Mantel coefficient | 0.266473 | 0.223847 | -0.02833 | 0.136861 | -0.10911 | 0.084986 | 0.129666 |
|  | p-value | 0.05 | 0.091 | 0.569 | 0.225 | 0.705 | 0.345 | 0.23 |
| Walnut oil | Mantel coefficient | 0.068184 | 0.172667 | 0.092493 | 0.114175 | 0.228466 | 0.022315 | 0.078302 |
|  | p-value | 0.335 | 0.149 | 0.29 | 0.258 | 0.075 | 0.444 | 0.322 |
| Sesame oil | Mantel coefficient | -0.1362 | 0.044035 | -0.12124 | 0.154341 | **0.372868** | 0.139457 | -0.00412 |
|  | p-value | 0.8 | 0.346 | 0.758 | 0.136 | **0.034** | 0.171 | 0.459 |
